# Polydopamine-Based Nanoprobes Application in Optical Biosensing

**DOI:** 10.3390/bios13110956

**Published:** 2023-10-27

**Authors:** Arianna Menichetti, Alexandra Mavridi-Printezi, Dario Mordini, Marco Montalti

**Affiliations:** Department of Chemistry “Giacomo Ciamician”, University of Bologna, Via Selmi 2, 40126 Bologna, Italy; arianna.menichetti2@unibo.it (A.M.); alexandra.mavridi2@unibo.it (A.M.-P.); dario.mordini2@unibo.it (D.M.)

**Keywords:** optical imaging, polydopamine, melanin, biosensing, fluorescent imaging, photoacoustic imaging

## Abstract

Polydopamine (PDA), the synthetic counterpart of melanin, is a widely investigated bio-inspired material for its chemical and photophysical properties, and in the last few years, bio-application of PDA and PDA-based materials have had a dramatic increase. In this review, we described PDA application in optical biosensing, exploring its multiple roles as a nanomaterial. In optical sensing, PDA can not only be used for its intrinsic fluorescent and photoacoustic properties as a probe: in some cases, a sample optical signal can be derived by melanin generation in situ or it can be enhanced in another material thanks to PDA modification. The various possibilities of PDA use coupled with its biocompatibility will indeed widen even more its application in optical bioimaging.

## 1. Introduction

**Scheme 1:**
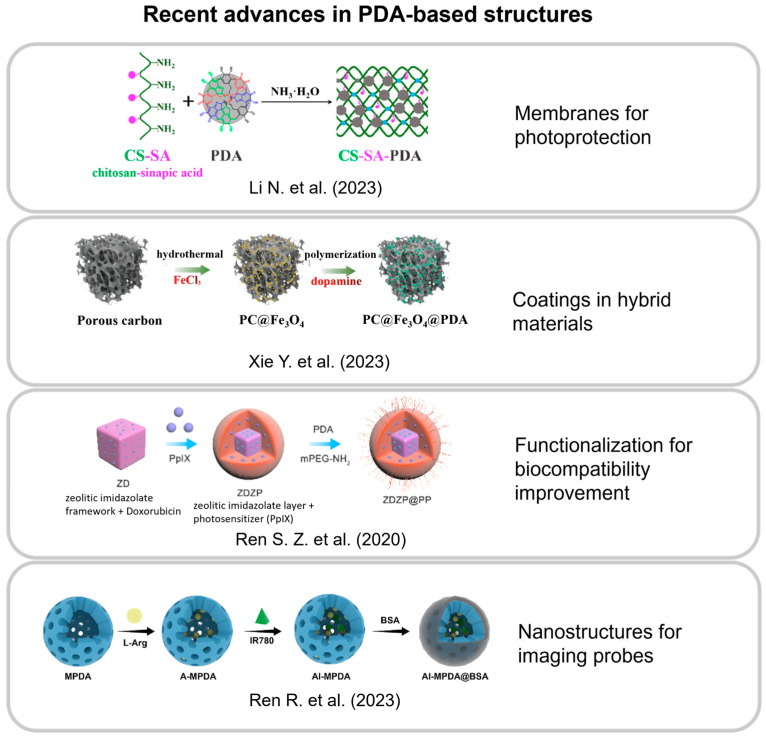
Examples of recent advances in PDA-based structures for photoprotection, hybrid materials and biocompatibility increase. Images from [36,44,48,51], with permission.

Optical bioimaging is one of the most investigated biosensing techniques, particularly studied in clinical diagnostics but also in food and environmental monitoring [1,2]. Indeed, it allows us to obtain signal generation with higher sensitivity, signal-to-noise ratio and stability with respect to other biosensing techniques [1,3]. For these reasons, in the last few years, many optical sensing techniques have been developed, such as fluorescence [4,5,6,7,8,9,10,11], chemiluminescence [12,13,14,15], surface-enhanced Raman scattering [16,17,18,19], localized surface plasmon resonance [20,21,22,23,24] and photoacoustic imaging [25,26,27,28]. Moreover, all these applications are usually developed using nanoprobes, given the extraordinary optical and surface properties of nanomaterials [29]. The most important requirements of optical biosensors are sensitivity, selectivity, and biocompatibility. Sensitivity and selectivity are strongly connected to the surface properties of the probe; indeed, its surface functionalities need to efficiently bind to receptors to induce responses [30]. Biocompatibility is also a challenging aspect, since nanomaterials are the most common probes used for optical bioimaging, their toxicity related to their small size (nanoparticles with size lower than 80 nm present higher translocation in the liver and if size is below 50 nm, cellular endocytosis is favored [31,32]). Their reactivity is a relevant factor for their applicability [33,34]. Ease of surface functionalization and biocompatibility can be jointly found in melanin and melanin-based materials. Melanin is a natural pigment produced in several living organisms, from mollusks to humans, with different roles, for example, in photoprotection [35,36] (Scheme 1) or in immune systems [37]. Polydopamine (PDA) is the synthetic analogue of melanin, produced from dopamine precursors that spontaneously polymerize in oxidative environments and basic pH [38]. One of the most intriguing properties of melanin-like chemical structures is the presence of functional groups, such as catechol, amine and imine, which can be a starting point both for molecules binding and metal ions anchoring [39] (Scheme 1). Indeed, PDA is widely used as a coating, which can effectively bind to inorganic substrates and then interact with other functionalities in the building of hybrid materials [40,41,42,43,44] (Scheme 1). The second prominent property of melanin and PDA is the excellent biocompatibility, which was largely studied and confirmed both in vitro and in vivo [45]. In particular, PDA is not only harmless to various cells [46] but also promotes their adhesion thanks to its surface properties [47]. Consequently, PDA has been demonstrated to enhance the biocompatibility of cytotoxic materials [48] (Scheme 1), such as semiconductor quantum dots (QDs) [49] or gold nanoparticles (NPs) [50].

**Figure 1:**
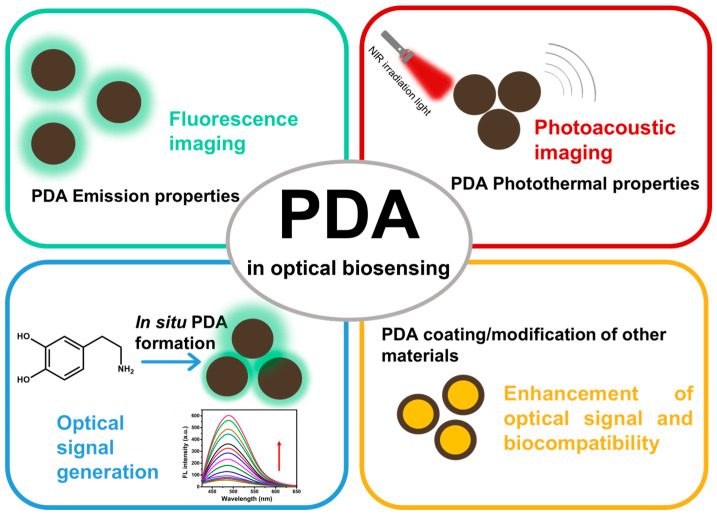
PDA roles in optical biosensing applications and dependence on its properties. Polydopamine fluorescence spectra graph from [64] with permission.

The ease of functionalization and the high biocompatibility described above have made PDA a perfect candidate for application in nanomedicine. Additionally, it possesses other important properties that have led to its use as a bioimaging probe (Figure 1). For example, PDA can be exploited in fluorescence signaling. It usually has a very low fluorescence efficiency, behavior correlated with melanin structure, and is composed of many aromatic oligomers that, because of π–π stacking, lead to aggregation-caused quenching (ACQ) [52]. However, the synthesis of polydopamine can be assisted by agents that enhance its fluorescence properties, controlling the polymerization and minimizing the aggregation between the oligomers: this has led to the development of fluorescent PDA nanoparticles [53,54]. Moreover, PDA can also behave as an effective fluorescence quencher, and this property is exploited as well in bioimaging [55,56,57]. Also, since, given its structure, non-radiative deactivation decay is normally favored, PDA is widely investigated for its high photothermal conversion efficiency [58,59]. Photothermal response is very useful for photoacoustic imaging (PAI), whose signal is based on thermoelastic expansion [60]. Many PDA-based nanosystems are emerging as PAI probes, often coupled also with therapeutic effects as photothermal therapy (PTT) agents [61,62,63]. In this review, PDA multiple roles and latest advances in optical bioimaging applications will be outlined (Figure 1).

This work will focus mostly on the papers of the last 3–4 years on PDA as a fluorescent and PAI probe, which are the main applications of this material in optical bioimaging. However, an optical signal can also be produced by the generation of PDA in situ, which will be described as another interesting use of this material. Finally, a paragraph is dedicated to the role of PDA in the enhancement of the optical imaging properties of other materials, highlighting the various potentials of the material in this field.

## 2. PDA-Based Fluorescent Probes

**Figure 2:**
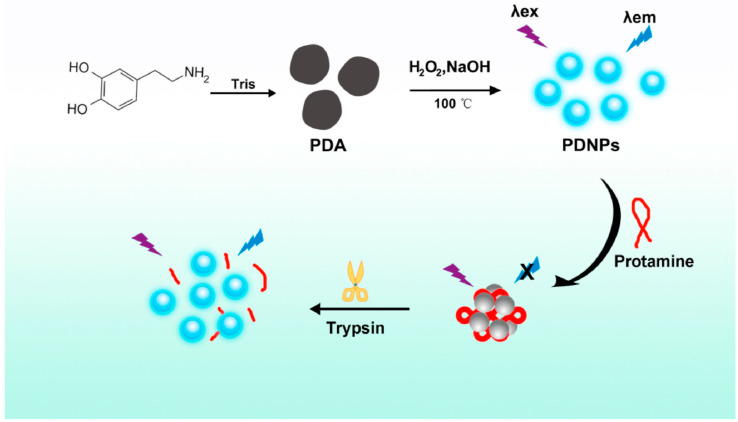
Mechanism of TRY detection by means of the fluorescence recovery of PDA NPs (PDNPs) after aggregation-caused quenching induced by Pro. Reproduced from [74] with permission.

Fluorescence imaging is one of the most developed techniques in biosensing, thanks to its high sensitivity, selectivity and spatiotemporal resolution [30] and its use is in continuous development for the detection of several bio-analytes [65,66,67]. Modification of PDA can lead to fluorescence properties, taking into account that the strategy to obtain fluorescent polydopamine is to reduce π–π stacking interaction, responsible for ACQ [52]. In particular, optimization of PDA fluorescence is obtained by the synthesis of polydopamine NPs performed by several methods, such as PDA oxidation, modification by ions and small molecules or surface stabilization by polymers [42]. It is worth noting that fluorescent PDA usually presents an emission that is excitation wavelength-dependent, probably due to nanoparticle size distribution or surface groups [68,69,70], and emission maxima strongly depend on the kind of precursor [71,72], so the photophysical properties of PDA NPs are always affected by starting materials and preparation methods. An example of the use of polydopamine NPs for biosensing is given by Tang et al., who developed a H_2_O_2_ and glucose sensor based on PDA–glutathione NPs [73]. These NPs (PDA-G(-S-) NPs) of 7–8 nm were obtained by the Michael reaction between GSH and dopamine under oxidative conditions, leading to an enhancement in NP fluorescence, with an emission maximum at 450 nm and a photostability of 90 min. At first, PDA-G(-S-) NP sensing towards H_2_O_2_ was investigated: a linear fluorescence decrease was observed in the presence of H_2_O_2_ in the concentration range 0.5–6 µM, with a detection limit at 0.15 μM, which is given by the oxidation of the thioether groups of the NPs. Then, since glucose oxidation, catalyzed by glucose oxidase (GOx), produces H_2_O_2_, PDA-G(-S-), NPs were also studied for glucose detection. A linear interval in the glucose concentration-dependent fluorescence decrease was found in the range 2.0–130 µM, with a detection limit of 0.6 µM, comparable to other fluorescent nanosensors. The selectivity of PDA-G(-S-) NPs was evaluated comparing their sensing ability towards glucose with other carbohydrates and metal ions, and finally, the system was also monitored in human serum, showing good applicability. Li and co-workers reported instead an “off–on” system based on fluorescent PDA NPs for the detection of trypsin (TRY), an enzyme connected to several diseases [74]. PDA fluorescent nanoparticles (ʎ_em_ = 435 nm, quantum yield of 2.2%) of approximately 5 nm were prepared by the oxidative degradation of PDA. Then, the interaction with protamine (Pro) was favored, leading to aggregation of PDA NPs and thus generating an aggregation-induced quenching. Pro is an arginine-rich protein that is hydrolyzed by TRY; as a consequence, in the presence of TRY, PDA NPs are disaggregated and their fluorescent signal is recovered (Figure 2).

This PDA-based fluorescent sensor gives a linear response for the TRY concentration range 0.01–0.1 µg/mL and a detection limit of 6.7 ng/mL. Moreover, the selectivity of the probe towards TRY was assessed testing the system with other proteases and ions, showing neglectable interferences. Fluorescence stability and resistance to photobleaching were investigated, resulting in a stable fluorescence signal in the pH range 3–10 after exposition to light illumination and after a storage of 50 days. Biocompatibility of the system was assessed by observing 92% A549 cell viability in the presence of a 3000-fold PDA NP concentration compared to that of the TRY detection assays. Fluorescent PDA was also used as probe for metal ion detection, exploiting the binding capability given by PDA surface groups, such as hydroxyl, amino or carboxylic groups. For example, Liu et al. and Li et al. reported the detection respectively of Zn^2+^ [75] and Cu^2+^ [76] ions. The former developed a fluorescent “off–on” system, exploiting the Zn^2+^ interaction with fluorescent (ʎ_em_ = 470 nm) PDA NPs (diameter up to 15 nm), obtaining a red-shifted enhanced signal (ʎ_em_ = 500 nm) upon binding with a linear trend up to 5 μM and limit of detection (LOD) of 60 nM. Li and co-workers, instead, did not use fluorescent nanoparticles, but synthesized dihydroxyindole oligomeric fluorescent derivatives (ʎ_em_ = 440 nm) for the detection of Cu^2+^ metal ions. In particular, they isolated oligomers that are formed during melanin polymerization, starting from melanin building blocks or their derivatives. These oligomers were tested with several metal ions, exhibiting the highest selectivity towards Cu^2+^ for the fluorescence quenching. This effect was more marked for the oligomer obtained starting from 5,6-dihydroxyindoles-2-carboxylic acid (P-DHICA); the most significant fluorescence quenching was obtained, probably caused by the stronger affinity of carboxylic groups for Cu^2+^. Fluorescence recovery is also observed after dissociation of Cu^2+^ (tested by the presence of pyrophosphate as competing agent), making P-DHICA a potentially reversible sensor. Apart from the detection of small molecules, ions and enzymes, fluorescent PDA NPs have also been studied for the recognition of cells or microorganisms, as in the case of the paper by Gu and co-workers, in which they were used in neuromast hair cell labeling in zebrafish lateral lines [77]. Fluorescent PDA NPs of tens of nanometers were obtained by the degradation of bigger PDA nanoparticles by the reaction with ethylenediamine (EDA). These PDA NPs exhibited a stable fluorescence intensity (ʎ_em_ = 525 nm), evaluated with irradiation cycles, and fluorescence lifetime of 4.7 ns, which was higher than typical fluorophores and allowed distinction between the sensor and cellular autofluorescence, enhancing the sensitivity. Biocompatibility was tested towards HeLa cells, resulting in 90% viability after 24 h of cell exposure to the NPs. These kinds of fluorescent markers are usually studied in cells in vitro, while in this work, interestingly, it was possible to evaluate the hair cell distribution in vivo during zebrafish larvae development. Fluorescent PDA NPs have been demonstrated to detect the hair cells efficiently and selectively, in a comparable manner with respect to typical hair cell fluorescent markers. Shen et al. developed a PDA-based fluorescence system for the detection of *Listeria monocytogenes* (*L. monocytogenes*), a pathogenic bacterium [78]. In particular, they obtained a nanozyme that could mimic peroxidase enzyme activity and act as a probe for a dual-mode detection. Fe-doped fluorescent PDA NPs (hydrodynamic diameter 62 nm) with emission wavelength 510 nm and a quantum yield of 15.1% were synthesized and then functionalized by aptamers to bind to *L. monocytogenes*. Their stability in water was assessed, denoting no change in fluorescence properties in the pH range 5.0–10.3. Direct proportionality between fluorescence intensity and logarithm of bacterial concentration was observed, in the range 3.0–1.0 × 10^7^ CFU/mL (bacteria per volume unit), with an LOD at 1.0 CFU/mL. The fluorescence assay was associated with a colorimetric assay obtained by the catalytic activity of the Fe-PDA NPs, which, by means of the formation of hydroxyl radicals in the presence of hydrogen peroxide, led to the oxidation of 3, 3′, 5, 5′-tetramethylbenzidine (TMB) to a blue (oxTMB).

**Figure 3:**
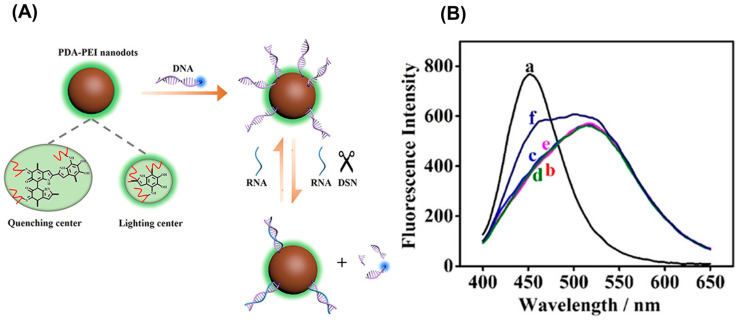
(**A**) Mechanism of miRNA detection by PDA–PEI nanodots integrated with AMCA-modified DNA. (**B**) Fluorescence spectra of (a) AMCA–DNA; (b) PDA–PEI nanodots; (c) PDA–PEI nanodots + DNA–AMCA; (d) PDA–PEI nanodots + DNA–AMCA + DSN; (e) PDA–PEI nanodots + DNA–AMCA + miRNA-21; and (f) PDA–PEI nanodots + DNA–AMCA + DSN + miRNA-21. Reported from [82] with permission.

In the examples described above, PDA-based systems were used in optical bioimaging as fluorescent probes. Nevertheless, PDA is also known for its efficient quenching capability that can be exploited for sensing applications [79]. An example of an optical sensor based on PDA fluorescence quenching is given by Yang and co-workers [80]. They presented a PDA–polypyrrole nanosheet (PDA–PPy NS) in order to efficiently quench a fluorophore-labeled DNA strand as a probe for a microRNA (miRNA-21), a tumor biomarker. The PDA–PPy NS was engineered in order to have the highest quenching efficiency, optimizing delocalization of π electrons and charge separation [81]. This nanosheet (15–20 nm of thickness) was then tested as a nanoquencher for nucleic acids by studying its interaction with a DNA single strand, labeled by the fluorescent dye Cy5. Absorption fluorescence spectroscopy revealed the successful association of the DNA strand and the fluorescence quenching of the Cy5 dye. This whole system was then used as an “off–on” probe for the detection of miRNA-21: it exploited miRNA-21 recognition by the fluorescence-labeled DNA strand, which, dissociating from the PDA–PPy NS, recovered its fluorescence. A PDA–PPy NS nanosensor has been shown to have a signal intensity linearly dependent on miRNA-21 in the concentration range 0.005–0.1 nM, with an LOD of 23.1 pM, lower than other biosensors such as PDA and GO nanoquenchers. Selectivity of a PDA–PPy NS nanosensor towards miRNA-21 was also confirmed by comparison with single-, double-, and three-base mismatched miRNA-21 and other miRNAs. miRNA detection was also successfully performed in fetal bovine serum as a first demonstration of the performances of PDA–PPy NS in real samples. Thanks to its fluorescence and quenching properties, PDA can also be used as a ratiometric probe, as described in the work of Deng et al. [82]. They synthesized nanodots (average diameter of 6.7 nm) from the copolymerization of PDA and polyethyleneimine (PEI). Thanks to their composition involving PDA and PEI segments, indole–PEI derivatives, PDA–PEI complexes, and PDA–PEI nanodots exhibited both fluorescence (ʎ_em_ = 522 nm, quantum yield 1–2%), quenching capability and stability of months; they were thus used as ratiometric probes for the detection of miRNA-21. In particular, nanodots were integrated with a DNA probe labeled with aminomethylcoumarin acetate (AMCA) with blue luminescence (ʎ_em_ = 452 nm), efficiently quenched when interacting with PDA–PEI nanodots. When miRNA-21 was bonded to the DNA strand on the nanodot surface, a duplex-specific nuclease (DSN) enzyme was added, which selectively digested the DNA in the DNA–RNA duplexes, recovering AMCA fluorescence (Figure 3A). The ratiometric response was obtained by excitation at a unique wavelength (365 nm), using the PDA–PEI nanodots’ fluorescence as a reference signal and monitoring the AMCA luminescence variation (Figure 3B).

MiRNA-21 sensing gave a linear relationship between concentration and emission intensity ratios of AMCA and nanodots (I_452_/I_522_) in the range 0.8–50 nM, with LOD at 0.52 nm. System selectivity was evaluated by using single- and three-base mismatched miRNA-21, miRNA-143 and a random sequence of miRNA, showing no significant interference. The biocompatibility of the PDA-PEI nanodots was also assessed by means of their uptake in HeLa cells, showing them to be cytotoxic to the cells and thus not suitable for in vivo cell imaging. The cytotoxicity of this system was attributed to the presence of PEI, which should be substituted in future developments of these nanodots.

In this paragraph, it can be clearly seen how PDA-based materials can be used in fluorescence as a quencher, but also as a fluorophore, despite PDA not being usually considered a fluorescent material. This is possible because of the existence of several PDA species, essentially based on the control of the degree of PDA polymerization or of PDA degradation. In this way, the fluorescent properties of PDA components and intermediates can be enhanced. However, looking at the works described above, a lack of high emission efficiency is observed. Indeed, quantum yield is often around 1–2% and in most cases it is not even reported, while it represents an important factor in determining the efficiency and sensitivity of the probe. Nevertheless, almost all the obtained imaging probes present good stability and photostability and quite high selectivity. The coupling of these properties with PDA biocompatibility widens the possibilities of its application in fluorescent biosensing.

## 3. PDA in Photoacoustic Imaging

**Figure 4:**
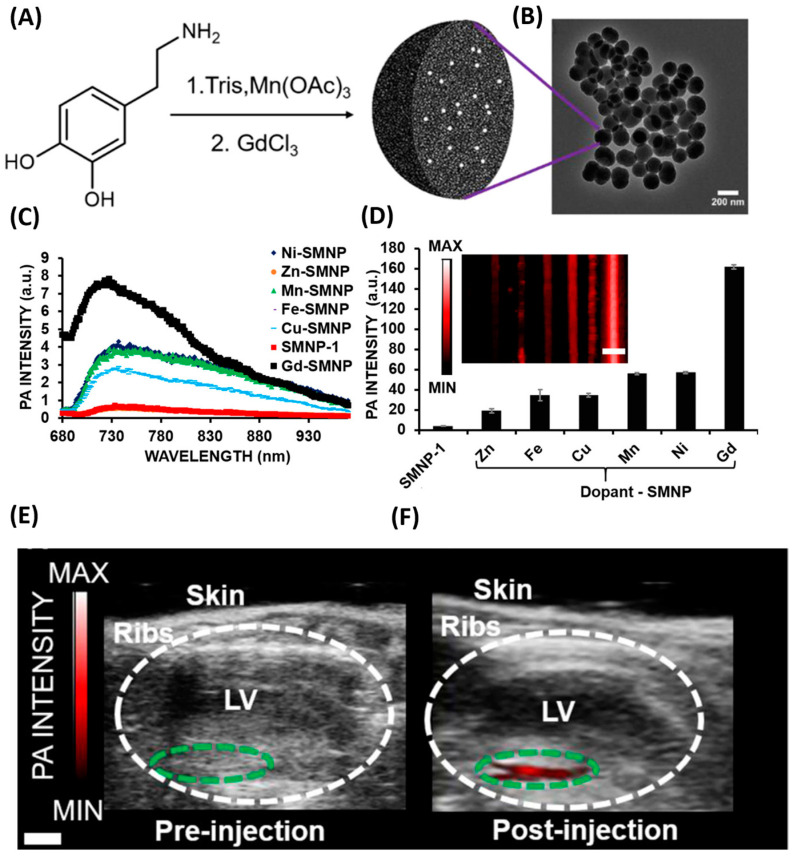
(**A**) Synthesis of Gd(III)-doped SMNPs and (**B**) TEM characterization. (**C**) Spectra of photoacoustic (PA) intensity in the wavelength range 680–970 nm and (**D**) PA intensity signals comparing Gd(III) and other ions (scale bar 4 mm), showing the best signal enhancement in Gd(III) doping. Photoacoustic imaging of the longitudinal axis view of live mouse heart preinjection (**E**) and postinjection (**F**) (scale bar 2 mm). White line circled area corresponds to the left ventricle (LV) and green line circled area to LV wall. Adapted from [86] with permission.

Photoacoustic imaging (PAI) is currently widely investigated in biomedical applications, because of its advantages with respect to other optical bioimaging techniques, such as low scattering of tissues, multiscale high resolution, high sensitivity and no background signal [83]. Photothermal agents are used as PAI probes and melanin-derived materials are the perfect candidates for this purpose, given their already discussed biocompatibility and their photothermal properties. In particular, PDA, due to its favored non-radiative deactivation pathways, presents a high photothermal efficiency, which has been more and more investigated in the last few years, mainly for nanomedicine purposes, such as photothermal therapy. Photothermal conversion performance can be improved by increasing PDA concentration and irradiation intensity [59]; another relevant parameter is the absorbance of PDA at a wavelength of irradiation that is usually about 800 nm, because of the use of NIR wavelength range in nanomedicine. Moreover, photostability is another very important property of PDA for photothermal applications [84], because these kinds of materials are supposed to be under continuous irradiation. In many cases, PDA is also modified by other ions or molecules to enhance its photothermal performance or to obtain multimodal and theragnostic systems. For example, Lin and co-workers developed a polypyrrole (PPy)–PDA based multimodal system, coupling Raman imaging and PAI [85]. They synthesized PPy–PDA hybrid materials, using SiO_2_ as a template and obtaining almost 200 nm core–shell SiO_2_–PPy–PDA NPs (SiO_2_−CS@PPy−PDA) with chondroitin sulfate (CS) to control the growth of PPy and PDA. The synergy between PPy and PDA was demonstrated to enhance both Raman and photoacoustic performances. In particular, SiO_2_−CS@PPy−PDA NPs had a photothermal conversion efficiency superior (40.7%) to that of PPy (29.6%) and PDA (20.1%) NPs. This is due to the bandgap decrease and photoinduced electron transfer between PPy and PDA molecules that enhance the non-radiative decay. Moreover, SiO_2_−CS@PPy−PDA NPs had higher absorbance in the near-infrared region compared to PPy, PDA and their mixture, increasing photoacoustic amplitude even more, and presented higher stability (they stay stable after 3.4 × 10^−4^ pulses) than gold nanorods taken as a reference. The system was tested both in vitro and in vivo. For the in vivo experiments, performed on BALB/c mice, no damage to the major tissues or liver/kidney disorders were observed. After biocompatibility verifications, tests were performed on tumor-bearing mice, in which a good contrast of the tumor tissue was observed with high spatial resolution, thanks to the combination of photoacoustic and Raman signals. Lemaster et al. reported the enhancement of PDA photoacoustic properties after doping with Gd(III) [86]. They obtained PDA nanoparticles (150 nm) with a high doping of gadolinium ions (Figure 4A,B), and they observed a dramatic increase in photoacoustic signal compared to metal-free NPs and also compared to the doping of PDA NPs with other metal ions (Figure 4C,D). The photoacoustic performance was given by the increased absorption of Gd(III)-doped PDA nanoparticles from 700 nm to 900 nm. Stability of the photoacoustic signal was verified by laser irradiation (680–970 nm) and biocompatibility was assessed by human mesenchymal stem cell (hMSC) viability, which were not influenced by the treatment with the NPs. Photoacoustic performance was evaluated in hMSCs, varying NP concentration, and monitoring the time of the treatment, and the most intense signal was for 0.42 mg/mL after 4 h. In vivo tests were performed injecting NP-labeled cells in mice to study myocardial infarction, showing a significant photoacoustic signal increase after injection (64-fold + 11.3) and thus demonstrating the PAI performance (Figure 4E,F).

Furthermore, Gd(III) being used in MRI, this technique was associated with PAI, obtaining a dual-mode system. Alternatively, Xie et al. obtained fluorine-modified polydopamine nanoparticles (80–140 nm) loaded with a US responsive perfluorocarbon volatile liquid (PFC) [87], initially for contrast-enhanced ultrasound (CEUS), and then they explored its performances also in PAI [88]. They synthesized PDA NPs with chelated Fe(III) ions, which were then fluorinated by functionalization of 1H, 1H, 2H, 2H-perfluorodecanethiol. Then, they were loaded with PFC. After having tested the CEUS performances, given PDA photothermal properties, the authors focused on the PAI behavior of the system. They noted that the PAI signal was enhanced in PFC-loaded nanoparticles, rather than in the unloaded ones, probably because of PFC-induced nanoparticle aggregation that generated an absorption increase. Moreover, PAI efficiency was increased by Fe(III) concentration, thanks to the charge transfer involving the Fe(III) chelating catecholate PDA moiety. The system was reported to be stable for 50 days and signal stability was also confirmed by exposing the system to 30 min of 700 nm laser irradiation. NPs were tested on HCT116 cells, showing good biocompatibility. Experiments in vivo were also performed analyzing chicken breast tissue, which resulted in good stability in time photoacoustic performances. Ma et al. also described a dual-mode imaging probe, using lanthanides to obtain second near-infrared (NIR-II) signals and PDA for PAI [89]. Lanthanide-doped downconversion nanoparticles (DCNPs) [90], core@shell@shell (CSS) NaYF_4_:Yb^3+^, Er^3+^@NaYbF_4_@NaYF_4_:Nd^3+^ were synthesized and coated with a PDA outer shell (DCNP@PDA NPs). These nanoparticles (with a diameter of almost 32 nm) presented good absorption properties in the NIR region and good photostability under laser (808 nm) irradiation, making them suitable for PAI application. Moreover, DCNP@PDA NPs showed negligible cytotoxicity towards NTH 3T3 cells. They were thus embedded in BALB/c mice for in vivo monitoring of the gastrointestinal (GI) tract, obtaining good contrast given by the coupling of PAI and NIR-II imaging. Peristaltic GI disorders were also mimicked to test DCNP@PDA NPs, which showed accumulation in the treated intestinal part, showing their suitability also for diagnosis. Other than in multimodal imaging, PDA-based PAI is often coupled with therapeutic effects. Indeed, since PDA often represents a support for the controlled release of other agents, it is very convenient to use it in a theragnostic system. For example, Ren and co-workers embedded agents for NO release and photothermal therapy (PTT) in mesoporous PDA (MPDA), exploiting its PAI efficiency [51]. MPDA was loaded with L-arginine as a NO donor and IR780 as a photosensitizer for ROS generation. Moreover, the whole system was covered by BSA to increase biocompatibility and biodistribution (AI-MPDA@BSA, hydrodynamic diameter of 220 nm). For PAI performance, the system had good absorption in the NIR region, 700–900 nm (given by the red-shift phenomenon involving aggregation of IR780 inside the NPs), which favored the irradiation by an 808 nm laser to obtain PAI. Photothermal effects of AI-MPDA@BSA were also evaluated, showing an increase in the photothermal efficiency given by the presence of IR780 and an NP concentration-dependent and laser power-dependent effect. Moreover, AI-MPDA@BSA NPs had good photothermal stability, tested with four “on–off” cycles of NIR irradiation. Cell viability of AI-MPDA@BSA NPs, evaluated in human umbilical vein endothelial cells, was reported to be close to 96%, even after 24 h of culture, indicating their biocompatibility. PAI was monitored in vivo by injection of the NPs in tumor-bearing mice, capturing images at different times up to 24 h. The PAI signal had its intensity maximum after 10 h from the injection, but was always visible over all the 24 h, demonstrating PAI efficiency in tracking NP in vivo distribution.

**Figure 5:**
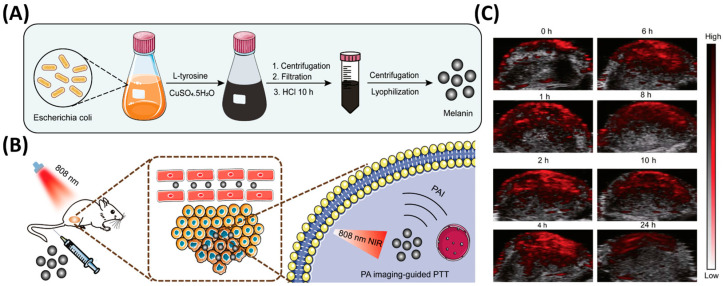
(**A**) Scheme of the synthesis of melanin NPs and (**B**) illustration of their action as PAI and PTT agents. (**C**) PA images of 4T1 tumor-bearing mice after intravenous administration of melanin NPs at different time intervals. From [94] with permission.

PDA photothermal efficiency is not just useful for PAI but also for PTT; indeed, in theragnostic application, PAI is often coupled not only with agent release, as described above, but also with PTT. Xiong et al. obtained theragnostic system-based non-PDA NPs doped by Cu^2+^ and embedded with a doxorubicin prodrug (HSD), named P(HSD–Cu–DA) NPs (size of 171 nm), that could associate chemodynamic therapy with PTT and PAI [91]. In this case, Cu^2+^, other than having a role in the chemodynamic therapy, contributed to the enhancement in the NIR absorption, favoring PDA photoacoustic performances (and also PTT ones). PAI was tested both in vitro, showing an increase in photoacoustic signal with NP concentration (irradiating at 744 nm) and a good photothermal stability (tested with 3 “on–off” cycles of laser irradiation), and in vivo after having verified P(HSD–Cu–DA) NP biosafety. In tumor-bearing mice, a strong photoacoustic signal was observed, denoting NP accumulation in tumor tissues. Analogously, Wu and co-workers embedded Mn_2_(CO)_10_ in MPDA NPs (hydrodynamic diameter of 260.8 nm) to obtain CO release in a tumoral environment and PTT, and observed nanoparticle distribution by means of MRI and PAI [92]. PAI was tested both in vitro and in vivo, showing good biosafety and intense photoacoustic signal. Pu et al. investigated an MOF nanosystem comprising PDA and Gd(III) ions to have dual-mode imaging (PAI and MRI) and to act as an antitumoral platform with PTT and PDT, the latter obtained by Ce6 loading [93]. They developed a core–shell structure of Gd–PDACe6@Gd-MOF (GPCG) NPs (hydrodynamic size of 183 nm), in which the PDA core serves both as photoacoustic and photothermal agent. GPCG NP biosafety was evaluated both in vitro (4T1 cells) and in vivo (mice), showing high cell viability and no damage to organs or tissues, respectively. GPCG NPs have been demonstrated to have an intense absorption and efficient photothermal effect under laser irradiation (808 nm) and good photostability under cycles of irradiation. Photoacoustic intensity was increasing linearly with the NP concentration and a bright signal was observed in tumor tissue of tumor-bearing mice, denoting its suitability as a PAI biosensor. Alternatively, Fu et al. managed to obtain theragnostic melanin NPs (size of less than 40 nm) just exploiting melanin photothermal conversion efficiency in PAI and PTT [94]. Rather than using PDA as in the cases described above, they obtained biosynthetic melanin by *Escherichia coli* tyrosinase gene (Figure 5A). Photothermal conversion efficiency was evaluated (by the method described in [95]), irradiating by a 808 nm laser, to be almost 48.9%, higher than PDA NPs, which are reported to have 27.7% efficiency. This outcome was in accordance with temperature increase measurements that showed better performances of biosynthetic melanin compared to PDA. Finally, photothermal behavior of biosynthetic melanin NPs was assessed by performing several irradiation cycles, remaining stable. Cytotoxicity of NPs in bEnd.3 and 4T1 cells was evaluated, resulting in 85% cell viability after 24 h of incubation, denoting their biocompatibility. Photoacoustic intensity was detected in vivo by injecting NPs in 4T1 tumor-bearing mice and at different time intervals (Figure 5C).

In particular, a photoacoustic signal was visible in the tumor tissue up to 24 h after injection, reaching the maximum at 2 h, and it thus led to a good tracking of the NP distribution for effective PTT. The recent literature cited above denotes the continuous increase in the use of PDA for PAI, with attention in increasing its photothermal efficiency and photostability and testing the systems in vivo. In particular, PDA performances in PAI are often enhanced by other elements. This could be caused by the need to increase the NIR absorption intensity or widen the NIR absorption window to obtain significant signal in the photoacoustic imaging. Indeed, tissue transparent wavelengths involve both the NIR-I (750–1000 nm) and the NIR-II (1000–1350 nm) windows [96,97], and thus there could be the necessity to boost melanin photothermal efficiency in those ranges. However, it is also worth noting that PAI is usually accompanied by other techniques in dual-mode and/or theragnostic systems. In these optics, the use of melanin is very beneficial, because it is a versatile platform for the doping or loading of dyes, ions and drugs, possessing at the same time the capability to both give PTT and generate photoacoustic signals. This makes it an essential component of theragnostic systems.

## 4. Optical Biosensors Based on Melanin and PDA In Situ Generation

**Figure 6:**
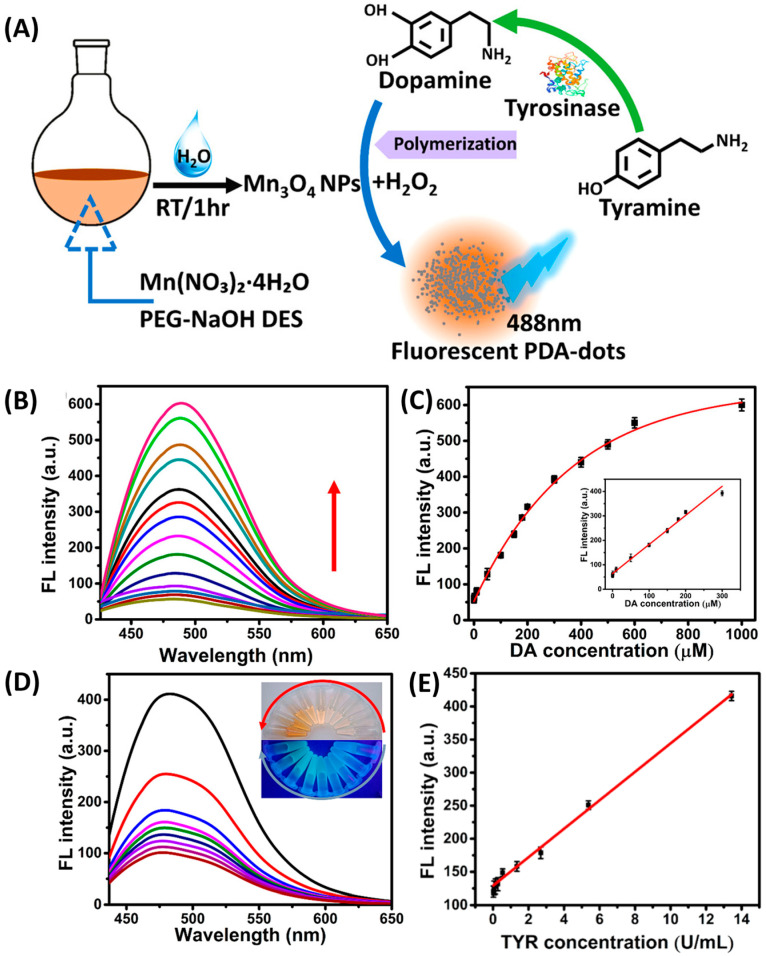
(**A**) Scheme of Mn_3_O_4_ NP synthesis and Mn_3_O_4_ NPs-induced polymerization of DA (obtained by tyramine and tyrosinase, green arrow) to fluorescent PDA dots (blue arrow). (**B**) Fluorescent spectra with increasing DA concentration and (**C**) fluorescence intensity relationship with DA concentration. (**D**) Fluorescent spectra with increasing TYR concentration (inset: increase of TYR concentration (red) connected to fluorescence increase (grey)) and (**E**) fluorescence intensity relationship with TYR concentration. Adapted from [64] with permission.

A recent application of melanin or PDA in biosensing is related to its generation in situ. Indeed, melanin formation is triggered by the tyrosinase activity and PDA is obtained by an oxidative environment, processes that can be of interest in the sensing applications. Also, as already mentioned in paragraph 2, fluorescence is generated by melanin–PDA synthesis intermediates and oligomers, which, by in situ polymerization, can be more easily monitored. In other cases, melanin or its precursors are simply not only the probe but also the target, as in the work of Tserevelakis and co-workers, who monitored melanin accumulation in fish scales, exploiting melanin PAI [98]. They implemented a hybrid confocal fluorescence and photoacoustic microscopy that could detect, by the combination of photoacoustic signal and tissue autofluorescence, the melanin content along the fish scales with high accuracy. Instead, PDA formation process was the way to detect dopamine (DA) in the work of Pang et al.; they monitored fluorescent PDA NPs (tens to hundreds of nanometers in diameter) formation by the peroxidase-like activity of ficin, a cysteine proteolytic enzyme, in the presence of H_2_O_2_ [99]. First, the method was verified and optimized by varying the experimental conditions. DA sensing was analyzed evaluating correlation with DA initial concentration and PDA NP fluorescence and was found to be linear in the DA concentration range 10 nM–5.0 µM, with an LOD of 5.5 nM. Selectivity in the DA probing was also assessed performing experiments in the presence of possible interfering agents in serum, such as ascorbic acid, urea, glucose, aspartic acid, lysine, alanine and phenylalanine, which confirmed no alteration of fluorescence signal even at high concentrations. Finally, the method was tested in human blood samples, resulting in recovery values of 92.5–110%, denoting applicability of the PDA NP formation in situ for biomedical purposes. Munyemana et al. developed a similar system, which aimed to detect DA and tyrosinase by means of the generation of fluorescent PDA NPs (average diameter of 1.7 nm) with Mn_3_O_4_ NPs as oxidants, in the presence of H_2_O_2_ (Figure 6A) [64]. After having synthesized Mn_3_O_4_ NPs, they studied their catalytic behavior in the production of PDA NPs, which was demonstrated to be efficient and quite stable. Afterwards, the fluorescence intensity of the PDA NPs was correlated with DA concentration and a linear dependence (Figure 6B,C) was reported for the DA concentration range 0.050–300 µM with LOD 0.017 µM. Also in this case, sugars, amino acids and cations were introduced to assess the selectivity of the fluorescence signal to DA. Additionally, the detection of tyrosinase enzyme was investigated, showing a linear relationship between fluorescence intensity and concentration (Figure 6D,E) in the range 0.021–13.43 U/mL and an LOD of 0.0048 U/mL.

**Figure 7:**
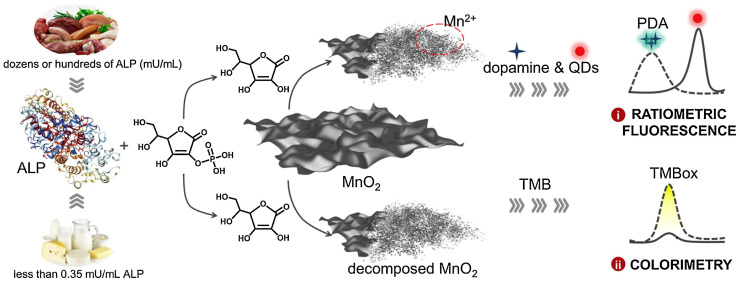
Ratiometric fluorescence and colorimetry dual-mode assay scheme of ALP activity. Reprinted from ref. [103] with permission.

Selectivity towards tyrosinase was assessed by comparison with other enzymes. Potential application of this system was also verified by means of tyrosinase detection in human blood serum, reporting good feasibility. Alternatively, Jesuraj et al. reported the detection of tyrosinase activity by means of absorption spectroscopy, exploiting the absorption spectrum variation during melanin formation [100]. They decided to correlate tyrosinase catalytic activity looking at the absorption increase at 475 nm at different time intervals. Indeed, that is the wavelength of the absorbance of dopaquinone and dopachrome, which are the principal melanin polymerization intermediates. The kinetics of tyrosinase were evaluated, testing the rate of dopachrome and dopaquinone formation at different enzyme concentrations and in the presence of an inhibitor, and the method was demonstrated to be an efficient tool to analyze tyrosinase activity. Other methods based on PDA formation monitoring were used to study the activity of alkaline phosphatase enzyme (ALP), which catalyzes the dephosphorylation of various substrates and is usually analyzed in livestock [101,102]. For example, Yang et al. developed an ALP activity biosensor with a ratiometric fluorescence assay based on fluorescence PDA generation and red-emitting quantum dots (QDs) [103]. They used MnO_2_ nanosheets to oxidize dopamine into PDA fluorescent NPs. PDA NPs quenched the red emission of CdTe/ZnS QDs because of fluorescence resonance energy transfer (FRET) and inner filter effect. Thus, under the action of MnO_2_, in the presence of dopamine and QDs, a green fluorescence signal increased (PDA NPs) and, at the same time, a red fluorescence signal decreased (QDs). The ALP activity towards molecules such as 2-phospho-L-ascorbic acid (AA2P) led to a reduction in MnO_2_ to Mn^2+^ ions, dopamine was no longer oxidated and fluorescent PDA formation failed, recovering QD red emission (Figure 7). This ratiometric fluorescent method was found to have a linear response for the 4–80 mU/mL ALP concentration range, with an LOD of 0.015 mU/mL when coupled with a TMB colorimetric method (Figure 7).

This technique was also investigated in livestock serum and milk samples, reporting a detection of ALP activity in the range 17.32–269.54 mU/mL. Nevertheless, this method was not selective if GSH or cysteine was present as reducing agent; however, the authors claimed that this interference could be blocked by a masking agent (N-ethylmaleimide—NEM) that could react with the thiol groups of the two molecules. ALP activity was also monitored in the work of Xue et al. by means of the same mechanism [104]. In this case, KMnO_4_ was used as the oxidizing agent for the synthesis of PDA fluorescence NPs (size 3.3 nm). ALP activity (in the presence of AA2P) was evaluated simply by means of the fluorescence decrease caused by the lack of PDA NP formation. Response linearity was found in the ALP concentration range 1–50 mU/mL with an LOD of 0.94 mU/mL. As concerns the selectivity, interference of GSH and cysteine was observed in this case as well. In this work, ALP activity was also monitored in the presence of an inhibitor and in human blood serum, since ALP can also be connected to bone diseases [105] and prostate cancer [106]: the applicability of the method on these samples was confirmed. Another example of ALP activity detection by means of PDA fluorescent NP generation is given by Li et al., which used CoOOH nanosheets as the oxidant agent [107]. Dopamine was oxidized by CoOOH, leading to the generation of fluorescent PDA NPs (5 nm); in the presence of ALP and AA2P, the AA obtained from ALP enzymatic activity reduced CoOOH and inhibited the formation of fluorescent PDA NPs. With this method, the range in which the linear correlation between ALP quantity and fluorescence intensity was 0.5–300 mU/mL, with an LOD of 0.1 mU/mL. Also in this case, ALP was then efficiently quantified in human blood serum. PDA in situ formation was also exploited for other analytes, such as in the case of Xu and co-workers, in which fluorescent PDA generation was used to detect pyrophosphate ion (PPi) and pyrophosphatase (PPase) activity [108]. FeCo-layered double hydroxide (FeCo–LDH) was synthesized as a peroxidase mimic, to catalyze, by means of the reaction with H_2_O_2_, the in situ oxidation of dopamine and obtain fluorescent PDA NPs. Then, in the presence of Fe^3+^, their fluorescence was quenched because of ion coordination. Fluorescence could be recovered with PPi that competed in Fe^3+^ coordination. After having optimized the conditions of the NPs’ synthesis and having verified Fe^3+^ effect on the fluorescence quenching, PPi detection was analyzed: a linear relationship between PPi and fluorescence increase was given for the concentration range 33–500 μM, with an LOD of 54 μM. Selectivity of this method was assessed by the comparison of the analysis in the presence of some phosphorus-containing anions and halide ions: these ions did not have significant interference with PPi detection. Other than PPi, also PPase activity was investigated; indeed, PPase hydrolizes PPi, freeing Fe^3+^ ions that bind again PDA NPs, quenching their fluorescence. Linear dependence of fluorescence could be demonstrated for PPase 0.17–3.33 mU/mL with an LOD of 0.13 mU/mL. Selectivity was assessed investigating the possible influence of molecules present in biological fluids, demonstrating good performances. A good correlation between the fluorescence signal and enzymatic activity was also found in human serum, denoting the feasibility of the system. Li and co-workers reported a biosensor based on PDA fluorescent NPs (with a size of 8 nm) formation in situ for the detection of four species: Fe^2+^, H_2_O_2_, DA and glucose [109]. They obtained dopamine polymerization by means of the Fenton reaction that, in the presence of Fe^2+^ and H_2_O_2_, generated the highly oxidant hydroxyl radicals [110]. Consequently, the authors exploited this synthesis as a sensing tool to correlate PDA fluorescence intensity with the reactants of Fenton reaction Fe^2+^ and H_2_O_2_ and DA. They investigated the sensing of each element separately from the others and optimized the reaction conditions to obtain the most significant signal variation. The recognition of Fe^2+^ showed two concentration intervals of linearity with fluorescence intensity, probably because of the number of hydroxyl radicals generated. An effective detection was possible in the ranges 0.5–10 μM and 300–100 μM, with an LOD of 0.09 μM. DA sensing had only an interval of linear response of fluorescence intensity, in the concentration range 0–300 μM, with an LOD of 0.07 μM. H_2_O_2_ gave a linear relationship with fluorescence in two concentration intervals, 1–60 μM and 100–500 μM, with an LOD 0.49 μM. Selectivity of the technique was also investigated. Fe^2+^ sensing comparison with other metal ions (such as Cu^2+^, Ag^+^, Zn^2+^) was conducted, demonstrating a significant selectivity. DA analysis was conducted with interference of other molecules and amino acids (such as arginine, cysteine, glycine), still obtaining good results. Analysis of H_2_O_2_ led to the possibility of the further detection of glucose, by means of GOx, since H_2_O_2_ is the one of products of the reaction of glucose with oxygen, catalyzed by GOx. Glucose linear relationship with fluorescence intensity was obtained for the concentration ranges 0–100 μM and 150–800 μM, with an LOD of 1.61 μM, comparable to other glucose detection methods. Glucose was also compared to other saccharides (sucrose, mannose, maltose and fructose), demonstrating high specificity towards its recognition. Tests were performed in human serum and a three-inputs molecular and logic gate [111] based on Fe^2+^, DA and H_2_O_2_ as inputs and fluorescence signal as output was also developed, demonstrating the practicability of this technique. Lee et al. used PDA formation control in situ to detect bacteria and their activity in the presence of antibiotics [112]. In alternative to most of the previous reported methods, they exploit the quenching capability of PDA formation rather that its fluorescence. They synthesized fluorescent dextran nanoparticles (FDNPs), which, in the presence of oxygen that initiates the polymerization of DA, were quenched by PDA. Moreover, the presence of amine groups on the FDNPs acted as a substrate of the polymerization process, making the fluorescence quenching even more effective. The idea was to sense bacteria because of their oxygen consumption, connected to fluorescence quenching inhibition. At first, quenching of FDNPs by PDA formation was verified by performing PDA growth measurements at different pH values and monitoring not only FDNP fluorescence but also DA polymerization by absorption spectroscopy. *Escherichia coli* (*E. coli*) was tested with this technique. In particular, the presence of 1.7 × 10^5^ CFU/µL *E. coli* consumed the oxygen in the environment, leading at least to the 75.9% fluorescence recovery; at 1.7 × 10^5^ CFU/µL the 99.5% of the initial fluorescence signal was recovered and PDA inhibition was also confirmed by absorption spectroscopy. In order to demonstrate that the fluorescence recovery was due to the lack of oxygen given by the bacterial activity, experiments were performed in conditions of bacterial minimal growth (4 °C incubation) and PDA formation was observed, demonstrating that the sensing process was not influenced by the surface cellular structure of the bacteria. Measuring bacterial activity allowed to also analyze their susceptibility to antibiotics. *E. coli*, negative or positive for NDM1, a gene involved in antibiotic resistance, was incubated in the presence of ampicillin. For the drug-resistant *E. coli* (NDM1+), a fluorescence signal was maintained, while for drug-sensitive *E. coli* (NDM1−), fluorescence was quenched, denoting *E. coli* death. These studies demonstrated how efficiently this method could be applied in the recognition of live bacteria growth. These overviews showed biosensing methods using not only melanin as in indicator, a fluorescent or PAI label or a quencher, but also exploiting its process of generation. Indeed, the elements that lead to PDA, such as enzymes, DA and oxidants, are all important in some biological functions and thus they can be considered not only as reactants but also as sensing targets.

## 5. PDA Modification of Other Optical Probes in Bioimaging

**Figure 8:**
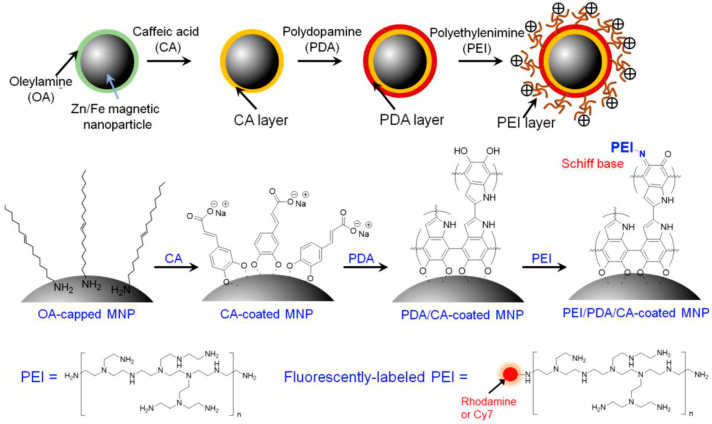
Scheme of the synthetic steps of the multifunctional nanoparticles based on Zn–Fe magnetic nanoparticles coated by PDA and fluorescently labeled PEI. Reprinted from [113] with permission.

In the last paragraphs, melanin–PDA systems were investigated for their use in optical bioimaging. Most of these applications are based on melanin intrinsic properties as a fluorescent probe or quencher and a PAI probe. However, in many cases, melanin is very useful as a support, such as a scaffold in which probes and drugs can be loaded or a coating for other nanosystems. This role that melanin often has can be related to the enhancement of the optical properties of the system or the increase of biocompatibility. For example, Kim and co-workers developed multifunctional nanoparticles to genetically manipulate natural killer cells and track them with magnetic resonance (MRI) and fluorescence imaging [113]. They synthesized Zn/Fe magnetic nanoparticles coated with PDA and with a cationic polymer (polyethylenimine PEI) labeled with an NIR fluorescent molecule (Rhodamine or Cy7) (Figure 8).

**Figure 9:**
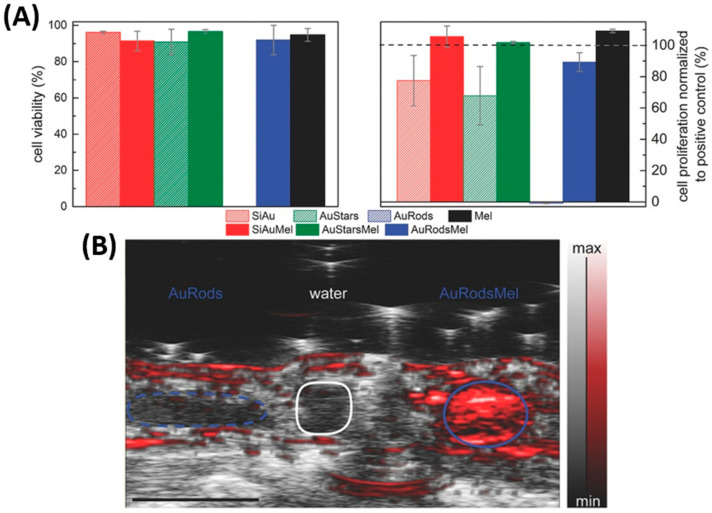
(**A**) Cell viability measured with live/dead staining and cell proliferation of L929 mouse fibroblasts incubated for 48 h. Particle concentration of 5.5 mg/mL with a particle concentration of 5.5 mg mL^−1^ after 48 h. Cell proliferation (normalized to no exposure of cells to gold NPs—dashed line) as a measure of cytotoxicity: L929 mouse fibroblasts are incubated for 48 h with a particle concentration of 5.5 mg mL^−1^. (**B**) Photoacoustic imaging signal of mouse intestine in the presence of AuRods (dashed blue), water (white), and melanin-coated gold nanorods AuRodsMel (solid blue) at concentrations of 12 mg/mL (scale bar 5 mm). Reported from [120] with permission.

The PDA coating was necessary to for the functionalization of the NPs with the labelled PEI. Indeed, PDA efficiently bonded PEI by a Michael addition or Shiff base [114,115]. Moreover, PDA enhanced the biocompatibility of the system, given the cytotoxicity of PEI [115]. Indeed, these NPs showed negligible toxicity towards NK-92MI cells, by which they were then genetically engineered. Strong fluorescence signals, obtained by the PEI labeled with Cy7 or rhodamine that were immobilized on the PDA surface, coupled with MRI, allowed tracking of NK cells with these multifunctional NPs. In the work of Jung et al., fluorescent nanodiamonds (FNDs) were encapsulated in PDA shell for bioimaging [116]. Indeed, FNDs, promising fluorescent probes, often tend to aggregate in physiological salt solutions and are difficult to functionalize with biologically active agents. For this reason, they were coated with a PDA shell: it allowed the functionalization with thiol terminated PEG, which prevents NPs aggregation and provides specific conjugation sites. This allows PDA–PEG-modified FNDs to be stable, even over a long time, in physiological salt solutions and to avoid nonspecific binding with the external cell membranes. Thanks to its specificity, further functionalization with biotine permitted it to act as a single-molecule fluorescent probe for DNA. The biocompatibility of this material was tested by cytotoxicity assays on mouse bone marrow-derived dendritic cells (BMDCs) and HeLa cells, showing no significant toxicity for either of the samples. Cell imaging was also significant, thanks to an effective uptake and efficient single-molecule tracking was also possible by the binding of the system with DNA. Hou and co-workers developed a very interesting bioimaging system based on the signal enhancement by means of FRET between plasmonic metal nanostructures and fluorophores [117]. Indeed, their interaction can lead to modification of the fluorophore properties, such as the enhancement of the emission efficiency by FRET [118]. These effect strongly depends, other than on the spectral overlaps, on the distance between the fluorophore and the plasmon metal structure [119]. In this work, the role of PDA was to control the distance between the plasmonic nanocrystals and the fluorophores, to maximize emission increase. Moreover, once coated on the nanocrystal surface, PDA allowed easy surface modification, and the PDA amphiphilic properties permitted the nanocrystals to self-assemble at the water–oil interface, making possible the formation and the transfer of particle 2D arrays. Au@Ag nanocubes, Au@Ag nanorods and Au nanorods were synthesized as plasmon metal nanocrystals to cover a wide spectral range. Then they were coated with PDA, whose shell, controlled at nanometer precision, was of 20 nm. Fluorescence enhancement was tested on the 2D array of plasmonic nanocrystals by three different dyes conjugated with BSA (which was needed to avoid aggregation-caused quenching or quenching caused by direct interaction with PDA). PDA thickness and good spectral overlap between plasmonic nanocrystals and the dyes led to the highest fluorescence enhancement with respect to other conditions. Other tests were performed using DNA molecular beacons with two fluorophores at the extremities, measuring their enhanced signal ratio and improving the sensitivity of the method from 2.89 × 10^−9^ M to 0.74 × 10^−9^ M of the target DNA. Finally, the FRET system was also successfully investigated on live cells (for epidermal growth factor receptor dimerization), after assessing its biocompatibility. Repenko et al. enhanced the photoacoustic signal of gold nanosystems by modification with PDA for bioimaging application [120]. Au NPs are indeed promising PAI agents, because of their absorption bands and their plasmon resonance. However, there are usually cytotoxic and their PAI efficiency is not so high, because of their poor absorbance in the range of tissue transparency. The role of PDA coating was both to improve the PAI performance of the pristine Au nanosystems and to make them biocompatible. PDA coating was obtained by auto-oxidative polymerization of DA on the surface of Au NPs of different shapes. PDA-modified Au NPs of every shape (NPs, nanostars and nanorods) displayed superior photoacoustic performances than pristine NPs. This behavior has been associated with PDA thermal expansion and thermal confinement [121]. In particular, PDA here acted as a thermal insulator, confining the heat in the AuNPs and leading to a higher thermal expansion, thus to a higher photoacoustic signal. As concerns the enhanced biocompatibility of the PDA-coated Au NPs, this was caused by the negative charge of PDA, which minimized the disruption of negative cell membrane. Moreover, Au NPs in the presence of PDA did not need stabilization in the biological medium, which was usually overcome by the addition of cationic surfactants, giving an added contribution to cytotoxicity. Cell viability of coated NPs (Au NPs, nanostars and nanorods) was tested and compared to the correspondent pristine Au NPs. Assay on pristine NPs led to cell viability of 85%, apart from the case of Au nanorods, in which cell apoptosis was observed. All PDA coated NPs demonstrated a cell viability of 90%, independently of the different shapes (Figure 9A). Finally, PDA-coated Au NPs were used as PAI probes in mouse intestine, showing highly stronger photoacoustic signal compared to pristine Au NPs, thus further demonstrating the PDA enhancement effect (Figure 9B).

These examples showed the role of melanin–PDA in the enhancement of the optical properties of other materials for optical bioimaging applications, exploiting PDA functionalities and biocompatibility.

## 6. Future Perspectives

In this review, the many roles of PDA-based materials in optical bioimaging have been described, denoting the versatility of this system. However, the properties of PDA-based materials, such as fluorescence and photothermal conversion efficiency, and the implementation of PDA-based materials as biosensors still need some improvements.

### 6.1. Comparison of Fluorescence and Photoacoustic Imaging Nanoprobes Based on PDA

Fluorescence and photoacoustic imaging are the biosensing applications in which PDA is undergoing its main development. Indeed, PDA has the versatility to be suitable for both techniques, even if its use can have advantages and disadvantages. Concerning fluorescence imaging, PDA biocompatibility and the ease of synthesis of fluorescent PDA NPs help to easily develop PDA-based nanoprobes. However, the often quite low quantum yield and the emission wavelength (that can lead to interference with background signal (REF)) can have an influence on the sensitivity and the selectivity. On the contrary, in PDA, photothermal properties are usually very efficient, allowing for a good sensitivity. Another advantage of PDA use in PAI is that, while PDA fluorescence can be obtained with visible light excitation, its photothermal activation occurs with NIR irradiation, allowing to PDA-based PAI probes to be observed also in vivo. However, PAI PDA performances are strongly influenced by light source intensity that in some cases need to be increased, leading to problematic and risky application in vivo. Considering these aspects, fluorescence and photoacoustic imaging are promising application for PDA-based nanomaterials, but in the future, they will need both to be increased in sensitivity and selectivity, in order to be more competitive to other biosensing materials.

### 6.2. PDA Fluorescence Control

Many biosensing systems exploit fluorescence of PDA. In particular, as already mentioned, PDA fluorescence can be obtained by PDA oxidation or synthesis of PDA NPs mediated by other molecules. However, fluorescence behavior of PDA is still not clear, because it comes from the fluorescence of intermediates and their oligomers in PDA formation [68]. This leads to a poor control on the fluorescence of synthesized PDA NPs that are usually used for bioimaging. Indeed, usually the fluorescence signal coming from these materials is excitation wavelength dependent [122], and the fluorescence wavelength reported in the papers in which PDA is used as a biosensor often corresponds to the one at which the emission signal is more intense and depends on the preparation methods of the fluorescent PDA. In these optics, there is no theoretical control of the fluorescence properties during PDA synthesis and fluorescence peaks have still not been assigned to PDA intermediates. However, it would be important to have a systematic investigation on the photophysical behavior of PDA intermediates and oligomers, in order to individuate the species responsible for each emission wavelength during the synthesis procedures. Indeed, the selection of a single wavelength-dependent fluorescence signal could be useful (i) to increase the intensity of the emission signal and (ii) to describe quenching events in a more precise manner.

### 6.3. PDA Control of Thermal Conversion in PAI and PTT

Thermal conversion is one of the main characteristics of PDA and it allows applications in PAI. Other than a diagnostic tool, PDA can also be useful for PTT, and thus photothermal properties give the possibility for PDA to provide theragnostic systems, combining PAI and PTT. In both applications, the light dosage, usually obtained by a laser in the NIR wavelength range, are particularly important. Indeed, in PTT the light irradiation must not be too powerful, in order to avoid damage of normal tissues [96] and PAI obviously involves a lower, not harmful dosage than PTT. For these reasons, other than controlling the light power, it would be important to have a control on the photothermal behavior. A deeper investigation on how PDA can be synthesized in order to increase its photothermal properties can help to combine PAI and PTT in a single system and respectively have the highest performances, lowering for both the applications the laser power, thus preventing collateral damages.

### 6.4. Development of Multifunctional Platforms Based on PDA Only

The PDA biosensors reported in the literature are often combined with other materials that are useful for the enhancement of the signal or to obtain multimodal imaging techniques. Considered PDA versatility, it would be interesting to develop multimodal and theragnostic systems based just on PDA, exploiting its functionalization potential, its fluorescent and PAI properties and its use in PTT. This possibility could become concrete only with an accurate control in the designing of PDA, in order to exploit its whole structure and at the same time all its components.

## 7. Conclusions

Optical biosensing is a field in continuous development because of the urgency to find effective diagnostic tools in nanomedicine. PDA comprises all the properties that an optical biosensor should have: (i) signal generation capability, (ii) ease of functionalization and (iii) high biocompatibility. These characteristics have directed the interest of researchers in many applications of PDA in optical biosensing, which are outlined in this review. PDA use in fluorescent sensors was reported, spacing from its intrinsic fluorescence properties to its quenching capability. Recent advances in PDA application in PAI was also described, considering its efficient photothermal conversion. Moreover, interesting application in bioimaging of PDA in situ generation was outlined, showing that in some cases, PDA represents not only the probe, but also the target analyte. Finally, we reported also the PDA capability to enhance the response of probes based on other materials, again denoting PDA functionalization potential. All these possibilities of PDA application in optical biosensing denote a growing interest among researchers and there is also a lot of space for improvement in this direction. Indeed, while there are no doubts about excellent PDA biocompatibility and ease of functionalization, its fluorescence and photothermal behaviors are still difficult to rationalize, because the mechanism behind it is still not completely outlined. For this reason, in the future, focusing on the intrinsic properties of melanin–PDA can surely help to trigger its use and competitiveness in the sensing field. Overall, this review highlights that in the field of optical biosensing, PDA is starting to be more and more present with many roles, and its versatility will surely lead to improvements and development of new optical probes.

## Data Availability

Not applicable.
